# Preparation of Composite Hydrogels Based on Cysteine–Silver Sol and Methylene Blue as Promising Systems for Anticancer Photodynamic Therapy

**DOI:** 10.3390/gels10090577

**Published:** 2024-09-05

**Authors:** Dmitry V. Vishnevetskii, Fedor A. Metlin, Yana V. Andrianova, Elizaveta E. Polyakova, Alexandra I. Ivanova, Dmitry V. Averkin, Arif R. Mekhtiev

**Affiliations:** 1 Department of Physical Chemistry, Tver State University, Building 33, Zhelyabova Str., Tver 170100, Russia; fedor-metlin@mail.ru (F.A.M.); nuri-chan-87@mail.ru (Y.V.A.); elizabeth03pol@gmail.com (E.E.P.); 2 Institute of Biomedical Chemistry, 10 Building 8, Pogodinskaya Str., Moscow 191121, Russia; 3 Department of Applied Physics, Tver State University, Building 33, Zhelyabova Str., Tver 170100, Russia; alex.ivanova33@yandex.ru; 4 Russian Metrological Institute of Technical Physics and Radio Engineering, Worker’s Settlement Mendeleevo, Building 11, Moscow 141570, Russia; averkindmitry@gmail.com

**Keywords:** L-cysteine, silver nitrate, methylene blue, low-molecular-weight gelators, self-assembly, photosensitizers, synergistic effect, reactive oxygen species, cancer, cytotoxicity

## Abstract

In this study, a novel supramolecular composite, “photogels”, was synthesized by mixing of cysteine–silver sol (CSS) and methylene blue (MB). A complex of modern physico-chemical methods of analysis such as viscosimetry, UV spectroscopy, dynamic and electrophoretic light scattering, scanning electron microscopy and energy-dispersive X-ray spectroscopy showed that MB molecules are uniformly localized mainly in the space between fibers of the gel-network formed by CSS particles. Molecules of the dye also bind with the surface of CSS particles by non-covalent interactions. This fact is reflected in the appearance of a synergistic anticancer effect of gels against human squamous cell carcinoma even in the absence of light irradiation. A mild toxic influence of hydrogels was observed in normal keratinocyte cells. Photodynamic exposure significantly increased gel activity, and there remained a synergistic effect. The study of free-radical oxidation in cells has shown that gels are not only capable of generating reactive oxygen species, but also have other targets of action. Flow cytometric analysis allowed us to find out that obtained hydrogels caused cell cycle arrest both without irradiation and with light exposure. The obtained gels are of considerable interest both from the point of view of academics and applied science, for example, in the photodynamic therapy of superficial neoplasms.

## 1. Introduction

The control and prevention of socially significant diseases is one of the most urgent tasks of our time [1]. Herewith, malignancies are an extremely difficult problem for society and the state as a whole. As an example, there are two million new cancer cases and more than half a million cancer deaths in the USA every year [2]. Among them, squamous cell carcinoma (SCC), belonging to a nonmelanoma-skin-cancer-related metastatic disease, is the second most common cutaneous malignancy [3]. There are several commonly accepted treatments for SCC, including surgical and nonsurgical procedures [4]. In the case of low-risk SCC, the latter approach can be used. Nonsurgical interventions include laser ablation, electrocoagulation, cryosurgery, local radiation and photodynamic therapy. The first four methods have disadvantages over photodynamic therapy (PDT) because of the lack of control of the histological margin and therefore the high probability of recurrence.

PDT is a modern, organ-preserving method of treatment for various diseases, especially gynecological ones. The technique is based on the use of photosensitizers (PSs)—substances sensitive to light and low-intensity laser radiation with a wavelength corresponding to the absorption peak of the photosensitizer [5,6]. As a result of this process, the generation of reactive oxygen species (ROS) occurs, which eventually leads to the death of cancer cells. PSs are divided into two large classes: transition metal coordination complexes and organic fluorophores. The former includes ruthenium (II) complexes [7,8], iridium (III) complexes [9,10] and polymetallic complexes [11,12]. Organic fluorophores as PSs have a long history of different applications, and compared to the metal coordination complexes, they are more effective under light exposure, less toxic and more biocompatible [13]. Among them are the following: naphthalene imides [14,15], xanthene dyes [16,17], porphyrins [18,19], phthalocyanines [20,21], boron dipyrromethenes [22,23], cyanines [24,25] and phenothiazine derivatives [26,27]. The latter, especially methylene blue (MB) and its derivatives, are one of the most promising PSs owing to low cost, small number of adverse effects, soft activation by ambient light and quick elimination from the body [28]. MB has shown the high efficiency in PDT against SCC [29,30,31]. However, the limitation of this dye in clinical practice is related to its rapid enzymatic reduction and conversion into the colorless leuco-MB form in the biological medium. In addition, MB molecule aggregation occurs in water medium due to the hydrophobicity of their structure, which leads to decreasing their activity in the generation of ROS.

The solution of the above problem is a formulation of different carriers based on organic (liposomes and polymers) and inorganic (quantum dots, ceramic-based nanoparticles, metallic nanoparticles and carbon materials) materials which prevent PSs from having premature loss of activity [32,33,34]. Hydrogels belonging in most cases to organic materials have attracted considerable attention as drug delivery systems due to their high loading capacity and biocompatibility [35,36]. The most interesting and effective among them, especially in photodynamic therapy, are photosensitive gels or “photogels” (PGs) [37,38,39]. The radiation, ultraviolet or visible light, causes alterations in the gel-network structure that promotes controllable drug release. For instance, cis/trans isomerization of the photosensitive azobenzene or its derivatives covalently linked with a polymeric chain can control the intrinsic properties of the sol–gel transformations [40]. Another example is the use of photocleavable o-nitrobenzyl, which leads to a site-specific degradation of cross-linked hydrogel matrices [41].

Recently, PGs based on low-molecular-weight gelators (LMWGs) have been developed [42,43] using the above-mentioned approaches. The materials obtained on their basis due to multiple non-covalent interactions between LMWGs combine the softness of the structure, the variety of nanostructured elements being formed, which determines their functions, and elegant properties when they work in tandem. There are several examples of LMWG-based gel fabrication for PDT [44,45]. It is known that some LMWGs can capture MB molecules and form stable gels [46,47,48,49,50]. However, there is no information on using any MB-loaded LMWG-based gel in PDT.

Amino acids and their derivatives are able to form a variety of nanostructures, which can be exploited for various biomedical applications [51]. Our scientific team has recently shown that supramolecular gels obtained based on L-cysteine and different silver salts possess a unique range of properties including anticancer [52,53,54,55], antibacterial/antibiofilm [56], photosensitive [57] and film-forming properties [58]. We believe that the combination of these properties, along with the proposed possibility of incorporation of an MB photosensitizer in the gel matrix, will allow us to develop a synergistic effect, which is extremely important for the purposes of PDT.

The current work aims to study peculiarities of supramolecular gel formation based on cysteine–silver sol (CSS) with MB and estimate their anticancer activity in vitro under visible light exposure. Stable gels were obtained in a wide concentration range of MB. MB molecules do not practically affect the morphological changes of the gel-network and its stability, while CSS particles do not suppress the photoactivity of MB. Obtained hydrogels have shown a synergistic effect against human squamous carcinoma cancer cells even without light irradiation. A mild toxicity of the gels was observed in normal human keratinocyte cells. The visible light exposure promoted a significant increase in the anticancer activity of gel systems. Thus, the novel composite “photogels” could have potential applications in photodynamic therapy.

## 2. Results and Discussion

### 2.1. Synthesis of Gels with Methylene Blue

**Figure 1:**
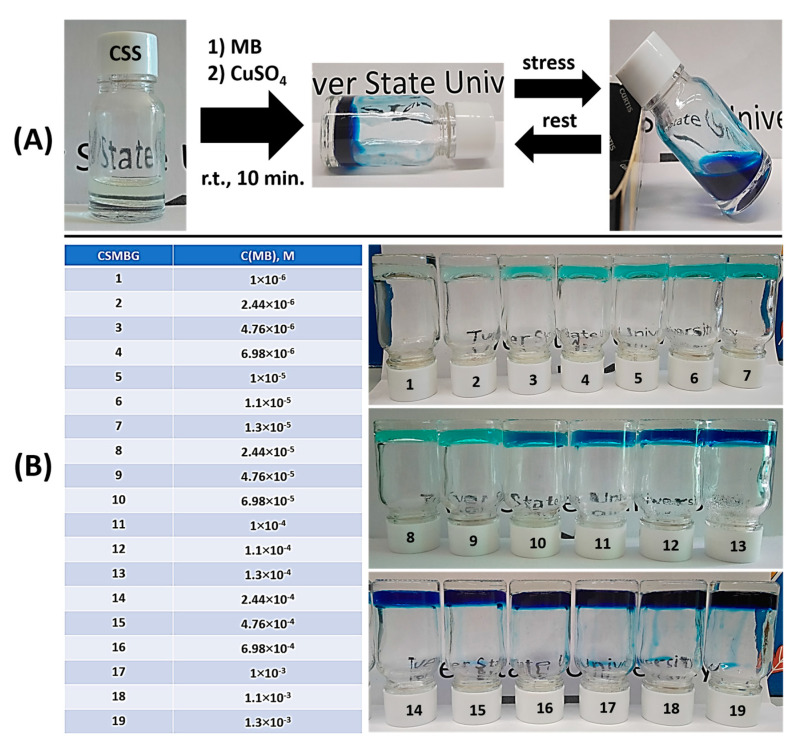
(**A**) The scheme of preparation of gels with MB. (**B**) The MB content in the gels (table) and photo of the gels. CSMBG—cysteine silver methylene blue gel.

The desired gels were synthesized in two stages (Figure 1A). CSS is a greenish-yellow-colored colloidal solution of nanoparticles which have a “core–shell” structure. The “core” is a crystalline phase of silver (AgNPs), and the “shell” is an amorphous phase of cysteine–silver complexes [53,54,56]. The sol stability is provided by the charged amino- and carboxyl-groups located on the surface of the particles. The total surface charge of the particle is positive. It has previously been found that various low-molecular-weight anions added to CSS are initiators of a gel formation [55,57,59]. Here, we have used copper sulfate (CuSO_4_) as an origin of anions. CSS and CuSO_4_ concentrations were fixed, and MB varied in a wide range (Figure 1B, table). One can see the formation of stable hydrogels (CSMBGs) at MB content from micromolar to millimolar concentrations (Figure 1B, photos). Herewith, no segregation or opalescence were observed, that is MB and CSS have a good compatibility. The obtained CSMBGs possess a thixotropic nature (Figure 1A). CSS nanoparticles have a positive surface charge value [52,53,54,55,56,57,58,59]; at the same time, MB is a cationic dye, and it would seem that it should destabilize the structure of a gel. Thus, in order to ascertain the peculiarities of the self-assembly in the system under study, a complex of physico-chemical methods of analysis was applied.

### 2.2. Physico-Chemical Analysis of Gel Formation

**Figure 2:**
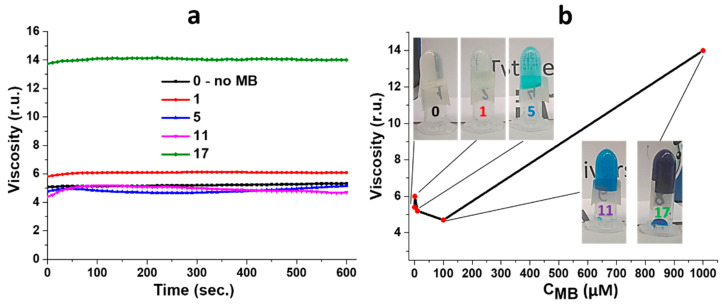
(**a**) Gel viscosity over time. (**b**) Gel viscosity dependence on MB concentration. See the sample numbers (1, 5, 11 and 17) in the table (Figure 1B).

Figure 2 demonstrates kinetic curves of gel viscosity and its dependence on the MB concentration. One can see that all curves immediately reach a constant value (Figure 2a). The viscosity of the gels varies slightly in the range of concentrations of MB up to 100 µM compared to the viscosity of a gel without a dye (Figure 2b). Herewith, a sharp jump in gel viscosity occurs upon increasing the MB concentration by an order of magnitude. Recently, a scientific team from Italy has investigated the influence of various dyes such as rhodamine B, disodium fluorescein, bromophenol blue and methylene blue on the rheological properties of gels obtained based on prolamin protein zein [60]. The authors have found that gel viscosity grows as the MB content increases. It should be noted that zein has a fairly similar surface structure (amino- and carboxyl-groups) to objects studied in this work. Thus, in order to understand the nature of interactions between MB molecules and CSS particles, the system should be considered at the micro- and nano-level.

**Figure 3:**
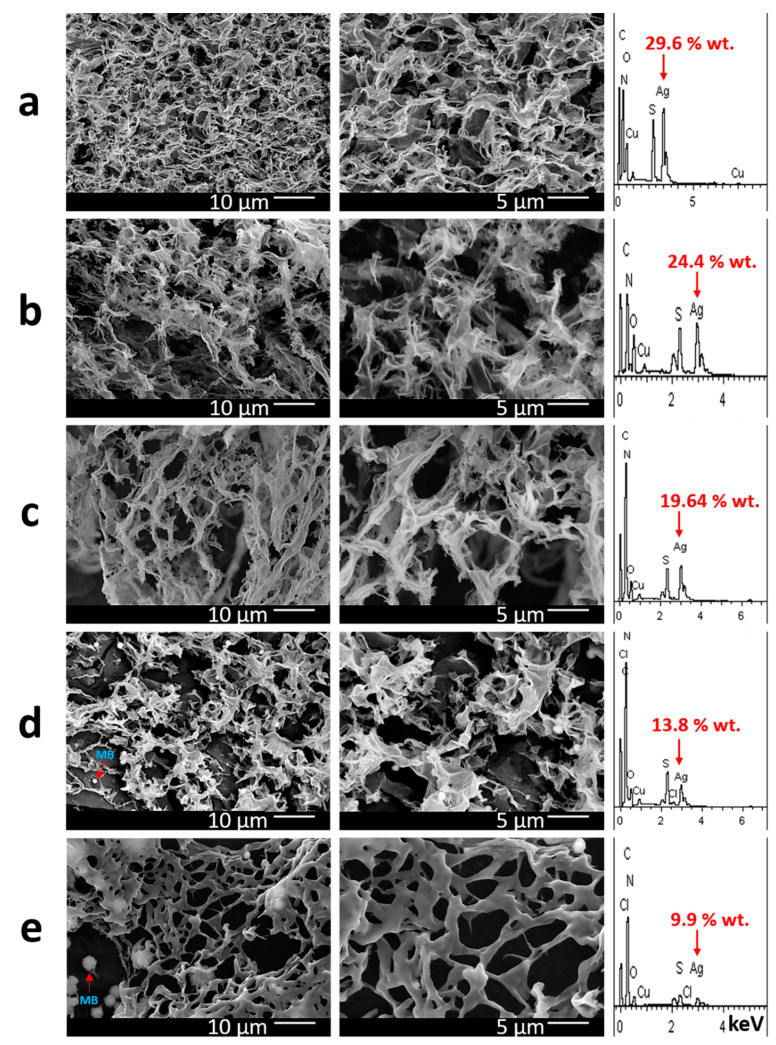
SEM images and EDS of gels: (**a**) no MB; (**b**–**e**) samples 1, 5, 11 and 17. See the sample numbers in the table in Figure 1B.

The morphology of the obtained gels is shown in Figure 3. The gel carcass is a ribbon-like fibrous structure. The gel without the MB has a quite dense gel-network (Figure 3a). The gel structure becomes sparser as the dye content increases. At the same time, the fibers of the gel-network thicken, and the structure of their surface changes from wrinkled to smooth when moving to the highest concentration of the dye (Figure 3e). The obtained results correlate quite well with the data of the viscometry analysis. Starting with the concentration of the dye of 10^−4^ M along with the fibrous structure of the gel-network, the presence of spherical motifs can also be noted (Figure 3d,e). The size of these particles grows as the MB concentration increases. Furthermore, these formations are incorporated both in the fibers of the gel-network and outside it. One can assume that these particles form due to the aggregation of MB molecules when the solvent is removed during the sample analysis. Indeed, MB is a crystalline compound, and it does not have a boiling temperature. The results of the elemental analysis confirm this supposition (Figure 3d,e): when the examined areas are scanned, traces of chlorine are found, which is a counter-ion in the MB molecule structure. Also noteworthy is the drop in silver content when moving to gels with a higher concentration of MB. Intermediate data indicate the possibility of interaction of MB molecules with the gel carcass, which forms from initial CSS nanoparticles [55].

**Figure 4:**
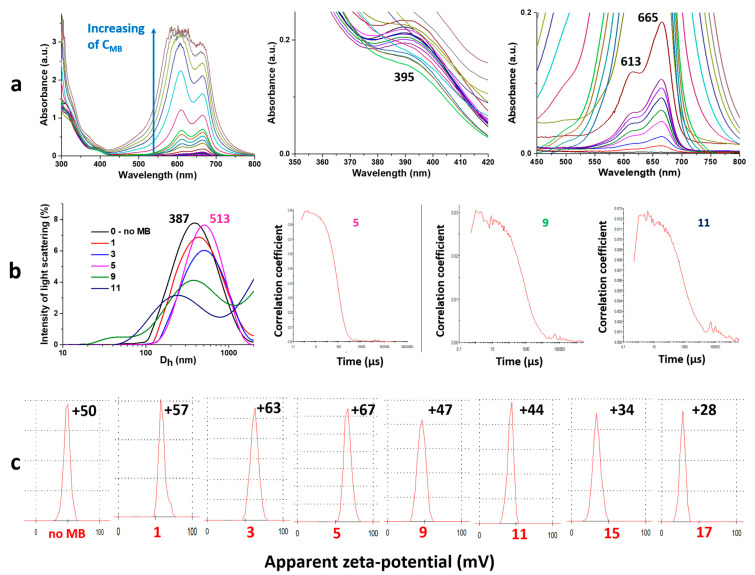
(**a**) UV spectra in various regions of wavelengths, (**b**) particle size distribution and cross-correlation functions and (**c**) zeta potential measurements for gels under study. See the sample numbers in the table in Figure 1B.

Due to the fact that the CSS and MB have characteristic absorption bands in the visible range of the spectrum, the UV method can provide some information about their interactions (Figure 4a). Bands at 310–320 nm and 390–410 nm correspond to the metallophilic interactions in cysteine/Ag^+^ complexes and surface plasmon resonance of AgNPs, respectively [52,53,54,55,56,57,58]. Absorbance at 613 and 665 nm is responsible for π-π* and n-π* electron transitions in the MB structure. One can clearly see that the electron spectra of both systems does not change upon CSS mixing with MB. This means the interaction between the initial structures is of a rather weak non-covalent nature. An increase in the dye content in the gel leads to the growth of the corresponding absorption bands. Since in this work there was no task of any quantitative analysis, we have given the results of the spectrum after the absorption value of 1, where the Bouguer–Lambert–Beer law is already non-linear. One can note that an increase in the dye concentration is reflected in a change of a 613/665 band ratio. It is well known that the band at 665 nm is associated with the monomer state of MB, and 613 with MB dimers [61]. The ratio of these forms does not exceed 1 up to a dye concentration of more than 10^−4^ M. This fact is very important in PDT, because the monomeric form is responsible for the high quantum yield of the present photosensitizer. The particle size distribution in resulting systems is monomodal (Figure 4b). The particle size gradually grows as the MB content increases. Herewith, starting with a certain concentration of the dye, the analysis of systems by the DLS method is impossible because the cross-correlation function has an irregular shape (Figure 4b, 9, 11 compared to 5). This phenomenon is due to the fact that the wavelength of the laser in DLS is 633 nm, which corresponds to the average maximum absorption of MB. Thus, instead of scattering, the dye absorbs almost all light. Finally, the dependence of the zeta potential of particles on the dye concentration has an extremum (Figure 4c).

Summing up the obtained results, one can propose the following self-assembly mechanism in the system under study: Mixing of MB with CSS leads to the formation of a homogeneous solution since MB molecules and CSS nanoparticles have the same surface charge; the addition of sulfate anions initiates the process of a gel formation; meanwhile, they interact mostly with the positive surface of CSS particles; MB molecules are located mainly in the space between formed fibers of the gel-network. With a low dye content in the system, MB molecules interact with the surface of CSS particles, which form the gel carcass, probably through weak ion–ion or ion–dipole interactions between the =S^+^ or =N^+^ site of the MB and COO^−^ or SO_4_^2−^ of the CSS particles. This is indicated by the DLS data and measurements of the zeta potential. At high concentrations of the dye, their molecules additionally start to displace and concentrate CSS particles due to the repulsion of similarly charged molecules, leading to the formation of larger aggregates. Herewith, the linear charge density drops on their surface. This is confirmed by the zeta potential measurements, SEM, EDS and viscosimetry analysis.

### 2.3. Anticancer Activity

**Figure 5:**
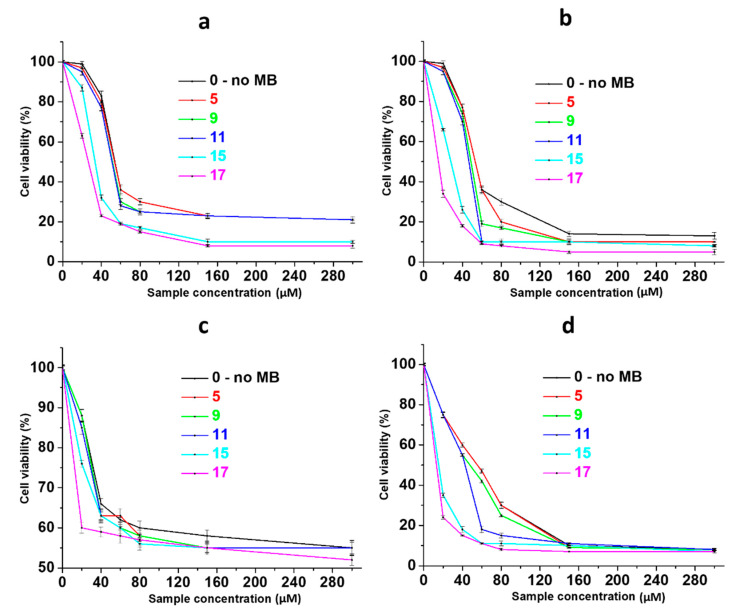
The cytotoxicity (MTT) of hydrogels for (**a**,**b**) SiHa and (**c**,**d**) HaCat cells without irradiation (**a**,**c**) and at a laser exposure of 638 nm (**b**,**d**). SiHa and HaCat cells’ incubation time with the systems is 48 h (n = 3). The information about the samples is given in the table in Figure 1B.

Hydrogels’ bioactivity to cancer and normal human cells is shown in Figure 5. In order to more correctly compare the activity of gels with MB (CSMBGs) and gel without dye, the concentration of particles in CSS was used along the abscissa axis in terms of the average content of L-cysteine and silver in the system. At the same time, the actual content of MB in samples for each concentration of CSS is shown in Table 1. One can see that all samples have a high toxic effect on SiHa cells even without laser exposure already at a concentration of 60 µM (Figure 5a). The incorporation of the dye into CSS significantly increases the effectiveness of composite gels (Figure 5a, 15 and 17). In this case, the gel activity is doubled at CSS concentrations of 150 and 300 µM, which indicates the additive effect of the system. Herewith, at a low concentration of CSS (40 µM), a synergistic effect is observed, since the toxicity of the system increases by 3–4 times. Indeed, according to the literature, the number of surviving SiHa cells is 80–90%, which is similar to the present work dye concentrations (4.9 and 10 µM) [62], and even at a concentration of MB of more than 150 µM, only 40% of cancer cells die. The cytotoxicity of all gels is approximately the same as HaCat cells, and it does not exceed their IC_50_ (Figure 5c). The laser irradiation of samples leads to an increase in gel activity by 2–3 times, which is especially typical for small concentrations of CSS (60 µM) and MB (2–20 µM) in gels (Figure 5b). And the synergistic effect remains. Here, healthy cells are significantly damaged even at low concentrations of the sample (Figure 5d). This fact should be taken into account during the PDT procedure, when the irradiation of the damaged area of the skin should be carried out pointwise.

**Figure 6:**
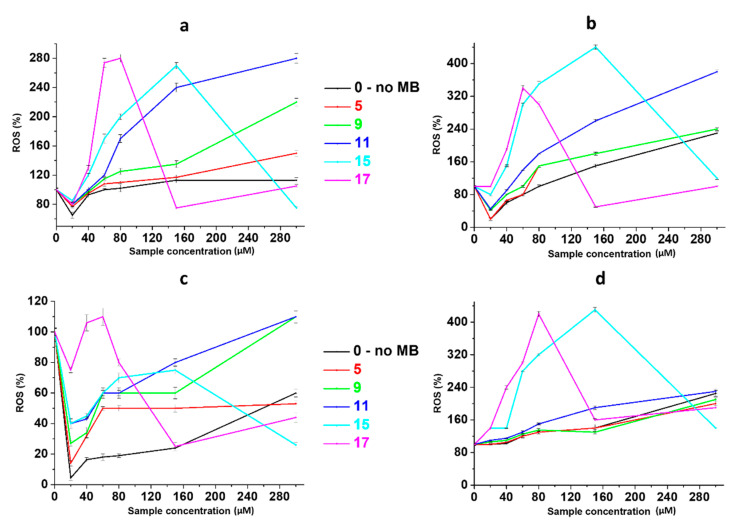
The ROS generation in (**a,b**) SiHa and (**c,d**) HaCat cells treated by hydrogels without irradiation (**a**,**c**) and at a laser exposure of 638 nm (**b**,**d**). SiHa and HaCat cells’ incubation time with the systems is 48 h (n = 4). The information about the samples is given in the table in Figure 1B.

MB generates cytotoxic reactive oxygen species during photoactivation, including singlet ^1^O_2_ oxygen. Figure 6 demonstrates data obtained during the study of the process of free-radical oxidation (ROS), occurring under photoactivation conditions in cells, during their incubation with CSMBGs. All systems are capable of ROS production even in the absence of irradiation (Figure 5a,c). Indeed, it is well known that AgNPs can cause oxidative stress without any external impact in various cells [63]. The higher the content of CSS particles and methylene blue, the stronger the effect. Herewith, a domed dependence is observed for samples 15 and 17, in which the dye content is higher than in the other samples. This may be due to the aggregation of MB molecules and the formation of its dimeric forms, which eventually leads to the effect of quenching incident radiation and reducing the yield of ROS. This fact is also confirmed by UV and DLS data. The average amount of ROS is 2–3 times higher in SiHa cells compared to HaCat cells for samples that have not been exposed to light (Figure 5a,c). This is probably due to the stronger capture of various materials by cancer cells in comparison with normal ones. During photodynamic exposure, the ROS content increases by 1,5–2 times in SiHa cells and 3–4 times in HaCat cells compared to unirradiated samples (Figure 6b,d). These results fully verify the MTT data. However, one can see that the curves obtained by these two methods (MTT and ROS) do not have symbatic dependence, which is probably connected to the partial oxidation of the dye by reactive oxygen species [57]. Furthermore, it should be noted that at low concentrations of the sample, a synergistic effect is not observed, but it occurs at higher concentrations. Thus, one can conclude that free-radical oxidation is not the only mechanism of cell death. This fact is extremely important for practical use, because the more targets of the drug’s action there are, the lower the likelihood that the pathological cells will become resistant to it.

**Figure 7:**
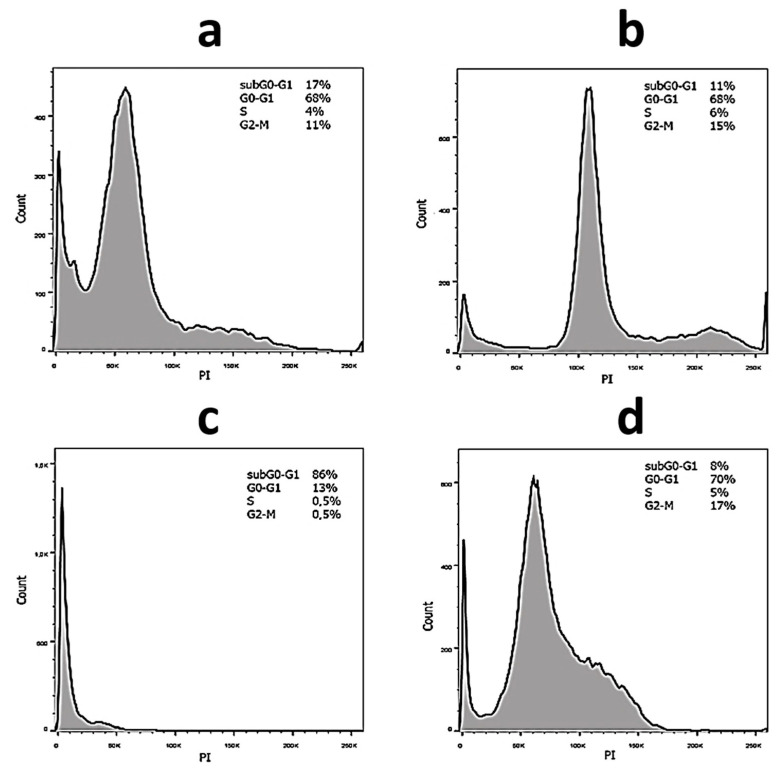
The flow cytometric analysis of DNA content in (**a**,**b**) SiHa and (**c**,**d**) HaCat cells treated by hydrogel (0—no MB) without irradiation (**a**,**c**) and at a laser exposure of 638 nm (**b**,**d**). SiHa and HaCat cells’ incubation time with the systems is 48 h. The concentration of the samples is 30 µM. The subG0–G1 peak on the histogram indicates apoptotic cells; G0–G1, S and G2-M—phases of the cell cycle.

Cell cycle arrest is the main indicator of the effectiveness of PDT in the treatment of cancer [64]. Typical diagrams of the distribution of the cell population of SiHa and HaCat cells, treated by hydrogels, in various phases of cell division are presented in Figure 7. Data on all samples are summarized in Table 2 and Table 3. The total number of actively dividing cells in the S and G2-M phases is approximately the same for all samples in the case of SiHa cancer cells (Table 2). One can see that the largest number of cells in the apoptosis phase (subG0–G1) was detected for MB-free gel both without irradiation and under exposure. However, all samples caused cell cycle arrest in the G0–G1 phase. Herewith, the incorporation of MB into the gel and increase in its content lead to this effect being strengthened by about 10%. The number of such cells practically does not change without irradiation as well as during photodynamic treatment. It is known that stopping the cell cycle in the G0–G1 phase is one of the ways to further cell death by the mechanism of apoptosis [65]. AgNPs [66] as well as MB [67] can lead to this pathway. The observed phenomena are probably associated with early and late apoptosis in the case of gels without MB and with dye, respectively. In the case of a normal cell line HaCat, their percentage in the apoptosis phase increases significantly compared to SiHa cells (Table 3). There is also cell cycle arrest in the G0–G1 phase. However, the content of cells in the subG0–G1 phase is significantly lower for gels with MB, while the number of dividing cells in the phases S and G2-M is higher, both in the absence of irradiation and under exposure. These facts are probably due to the different nature of these cell lines, as well as the physico-chemical properties of the systems observed without and under irradiation.

To sum up, one can assume that both without irradiation and under PDT, the synergistic effect of CSMBGs on cancer cells is related to the possibility of a supramolecular complex formation between CSS nanoparticles and methylene blue molecules, which was confirmed by a number of physico-chemical methods of analysis. At the same time, it is necessary to carry out further experiments both to clarify the self-assembly process and ascertain exact mechanisms of action of synthesized composite gels on cell lines.

## 3. Conclusions

In conclusion, novel supramolecular photoactive hydrogels based on CSS and MB have been synthesized for the first time. The formation of stable gels proceeds in the MB concentration range from 10^−6^ to 10^−3^ M. The gels’ viscosity changes with a sharp jump as the dye concentration is increased. The fibers of a gel-network thicken, and the structure of their surface changes from wrinkled to smooth only for the highest concentration of MB. MB molecules tend to interact with the surface of CSS nanoparticles, while they do not affect the stability of the gel and the suppression of the activity of the dye. Obtained “photogels” showed a synergistic effect of squamous cell carcinoma suppression with mild toxicity to keratinocyte cells at a micromolar concentration of CSS (60 µM) and MB (10 µM) without light exposure as well as during photodynamic treatment. Hydrogels have several targets of action on cancer cells, including the possibility of free-radical oxidation even in the absence of light irradiation. Systems are able to arrest the cell cycle due to its blocking in the G0–G1 phase both without light exposure and during PDT. Future studies will be related to understanding the relationship in the chain “structure–property” via additional instrumental techniques aimed at ascertaining interactions of CSS with MB and mechanisms by which gels influence the cells.

## 4. Experimental Section

### 4.1. Materials

L-cysteine (>99%) was supplied by Acros. Silver nitrate (>99%) was obtained from Lancaster (Lancashire, UK). Methylene blue (pure) and copper sulfate (pure) were purchased from Agate-Med (Moscow, Russia). All chemicals were used with no further purification. De-ionized water was used for the preparation of the systems under study.

### 4.2. Synthetic Protocol of Gel Preparation with Methylene Blue

A volume of 2 mL of hydrosol based on L-cysteine and silver nitrate (CSS) was synthesized in accordance of our previous report [53]: 0.6 mL of L-cysteine aqueous solution (0.01 M) was poured into an empty vessel and further diluted with 0.65 mL of the distilled water, then 0.75 mL of silver nitrate aqueous solution (0.01 M) was added. L-cysteine/AgNO_3_ = 1:1.25. The obtained opalescent mixture with a white-yellow shade was stirred for 1 min at room temperature (25 °C) and left for storage in a dark place for 3 h. A transparent hydrosol with a greenish-yellow color was obtained. Hydrogels were prepared in two stages. Firstly, the methylene blue aqueous solution of various concentrations was added to CSS, and the mixture was stirred for 1 min. Secondly, 0.09 mL of CuSO_4_ (0.01 M) was added to this mixture, and the solution was stirred for 1 min. As a result, after 10 min, transparent hydrogels (CSMBGs) with various final concentrations of MB (see table, Figure 1B) were obtained and stored in a dark place at room temperature.

### 4.3. Viscosimetry

The viscosity measurements of hydrogels were performed using an SV-10 (A&D, Tokyo, Japan) vibratory viscometer. The frequency vibration of sensor plates was 30 Hz with a constant amplitude of about 1 mm. Special polycarbonate cups (A&D, Tokyo, Japan) were used for the preparation of 10 mL of investigated systems. Samples were kept in a dark place for 24 h. After that, these cups were transferred to the viscometer, and the measurements were recorded at 25 °C.

### 4.4. Scanning Electron Microscopy and Energy-Dispersive X-ray Spectroscopy

A JEOL 6610 LV electron microscope (JEOL Ltd., Tokyo, Japan) with the Oxford INCA Energy 350 X-ray energy-dispersive microanalysis system (JEOL Ltd., Tokyo, Japan) were used for the study of the morphology and elemental composition of the samples. All experiments were carried out using a high-vacuum mode with an accelerating voltage of 15 kV. Low-energy secondary electron signals, providing topographical contrast, and high-energy back-up scattered (reflected) electrons, which determine the composition and phase contrast, were generated for the image acquisition. X-ray spectral microanalysis related to the registration and analysis of the energy spectra of the characteristic X-ray radiation excited by electrons passing through the sample was performed for the determination of the elemental chemical composition of the samples. The preparation of the samples included spraying them onto the thin conductive layer of a platinum surface and drying them in a vacuum (10^−4^ Pa). The average platinum coating time was 5 min.

### 4.5. UV Spectroscopy

The recording of the electronic spectra of the samples was performed by a UV spectrophotometer Evolution Array (Thermo Scientific, Waltham, MA, USA) in a quartz cell with 1 mm of an optical path length.

### 4.6. Dynamic Light Scattering

The size of particles formed in the systems under investigation, as well as their zeta potential, was measured via a Zetasizer Nano ZS (Malvern, Worcestershire, UK) equipped with a He-Ne laser (633 nm), with a power of 4 mW. Hydrogels were transferred into a sol state by shaking and then diluting them two, four and eight times. All measurements were carried out at 25 °C in the backscattering configuration at an angle of 173°, which provides the highest sensitivity of the device. The mathematical processing of the obtained results was performed using Zetasizer Software v7.11. The scattered light intensity values were measured after a time interval τ, and the so-called cross-correlation function *g*_2_(*τ*) was obtained (1):

(1) $$g_{2}\left(\tau \right)=\frac{\langle I(t)∙I(t+\tau )\rangle }{\langle I^{2}\rangle }\;$$

The value of is at its maximum when the value of *τ* is small compared to the lifetime of the concentration fluctuation, and *g*_2_(*τ*) = 0 when *τ* is much longer than the lifetime of the fluctuation. Thus, the value of *g*_2_(*τ*) attenuates with an increase in *τ* from the maximum value to zero. The equation of dependence of this *g*_2_(*τ*) function on the diffusion coefficient *D* has the following form (2):

(2) $$g_{2}\left(\tau \right)=1+C{\left[\int_{D_{min}}^{D_{max}}Z\left(D\right)\exp\left(-q^{2}D\tau \right)dD\right]}^{2}\;$$

*Z*(*D*) is the distribution function of scattering particles by the diffusion coefficients. This equation was solved by the cumulant method. As a result, the *Z*(*D*) function was obtained. An average *D* was calculated from the attenuation time—the time when the function decreases by e (2.74) times of the cross-correlation function. An average hydrodynamic radius of the scattering particles was calculated from the diffusion coefficient by the Stokes–Einstein formula: D = kT/6πηR, where D is the diffusion coefficient, k is the Boltzmann constant, T is the absolute temperature, η is the viscosity of the medium and R is the radius of the scattering particles.

### 4.7. Electrophoretic Light Scattering

Electrophoretic mobility measurements of particles in samples were carried out in U-shaped capillary cuvettes. Hydrogels were transferred into a sol state by shaking and then diluted two, four and eight times. Distributions of the zeta potential were calculated using the Henry equation: UE = 2ezf(Ka)/3Z, where UE—electrophoretic mobility, z—zeta potential, e—dielectric constant, Z—viscosity and f (Ka)—Henry’s function; f (Ka) = 1.5 for aqueous media.

### 4.8. Cells and Culture

Commercially available standard human cell cultures were used: human cervix carcinoma cells SiHa were obtained from the American Tissue and Cell Collection (ATCC, Catalog No.: HTB-35^TM^, Manassas, VA, USA) and HaCaT (a spontaneously transformed aneuploid immortal keratinocyte cell line from adult human skin) was obtained from Amsbio Company (Catalog No.: AMS.CL-0090, Milton, UK). Cell lines were cultured in 25 cm^2^ Corning flasks in 4 mL of DMEM (Dulbecco’s modified Eagle medium) (PanEco, Moscow, Russia) with 10% FBS (fetal bovine serum) and antibiotics, penicillin concentration 50 units/mL and streptomycin 50 μg/mL (PanEco, Moscow, Russia). Cells were transplanted and split 2–3 times a week. For detachment, the monolayer was treated with a solution of Versene (PanEco, Moscow, Russia), then with a solution of trypsin (Gibco, Waltham, MA, USA). Trypsinolysis was inhibited by DMEM culture medium with 10% serum content. The cell count was performed using Vi-CellTMXR (Beckman Coulter, Brea, CA, USA). For cell counting, a single cell suspension was prepared in a ratio of 1:10 (100 µL of cell suspension, 900 µL of medium).

### 4.9. Cell Photodynamic Treatment

The HaCat and SiHa cells were cultured in a medium containing CSMBGs at various concentrations for 24 h in the dark. The HaCat and SiHa cells incubated with the samples were subjected to photoexposure using an LED phototherapeutic device (Troitsk, Russia) (wavelength λ = 638 nm, power 270 mW); the irradiation dose was 1.8 J/cm^2^, and the treatment time was 10 min. After irradiation, the cells were cultured in the dark at 37 °C under 5% CO_2_ for 48 h.

### 4.10. Cytotoxicity Evaluation (MTT Test)

Cell lines were cultivated in 96-well plates at 37 °C under 5% CO_2_ in a DMEM medium supplemented with the addition of L-glutamine (2 mM), antibiotics (50 units/per mL of penicillin and 50 μg/mL of streptomycin) and 10% of FBS. The cells were incubated in a serum-containing medium with the tested compounds (CSMBGs) at various concentrations for 48 h. PBS (phosphate-buffered saline) at a volume of 10 μL containing MTT (3-(4,5-dimethylthiazol-2-yl)-2,5-diphenyl-tetrazolium bromide) 5 mg/mL was added to each well, and the cells were incubated at 37 °C for 4 h. The culture medium was removed, 100 μL of DMSO (dimethyl sulfoxide) was added to each well, the plate was vortexed for 20 min and then the optical absorbance in each well was measured at 570 nm by a Multiskan Spectrum Microplate Reader instrument (Thermo Scientific, Waltham, MA, USA). The MTT test readings were averaged for three independent measurements. Readings of the MTT test in the absence of the tested compounds were taken as 100%.

### 4.11. ROS Detection by 2′,7′-Dichlorodihydrofluorescein Diacetate (H2DCFDA)

The cells were seeded in 96-well plates and cultured at 37 °C in a humidified incubator supplied with 5% CO_2_. After 24 h, samples (CSMBGs) at various concentrations were added to the cells, and the systems were incubated for 48 h. To detect ROS in cells after the addition of samples, unfixed cells were stained with 2 μM 2-(3,6-diacetyloxy-2,7-dichloro-9H-xanthen-9-yl) benzoic acid (H2DCFDA) (Lumiprobe, Moscow, Russia) solution for 2 h at 37 °C in darkness. Then, the cells were carefully washed with PBS 2 times for 5 min. The obtained specimens were analyzed using a Tecan Genios PLUS Microplate Reader (Thermo Scientific, Waltham, MA, USA) with fluorescence at 485 nm and 535 nm, respectively. The ROS test readings were averaged for four independent measurements. Readings of the ROS test in the absence of the tested samples were taken as 100%.

### 4.12. The Cell Cycle Analysis

The cells were suspended in DMEM culture medium supplemented with 10% FBS and placed seeded in 6-well plates (at a concentration of 10^6^ cells/well). After the cells adhered, the tested compounds (CSMBGs) were added to wells at 100-fold dilution. The cells were irradiated. After 48 h post-treatment, the cells were washed with PBS and then harvested using Versene solution and 0.25% trypsin. Then, the cells were centrifuged at 250× *g* in PBS for 3 min twice. Washed cell pellets from each well were resuspended in a small volume of PBS and fixed with 0.5 mL of 70% ethanol for 30 min. Fixed cells were incubated with 200 μL of RNase A (200 μg/mL) solution in PBS supplemented with 0.1% *v*/*v* Triton X-100 for 30 min in the dark at 4 °C for 45 min and finally stained with 100 μL of 1 mg/mL propidium iodide (PI) at 20 °C for 10 min. The fluorescence of the stained cells was measured using a flow cytometer BD FACSAria III (BD Biosciences, East Rutherford, NJ, USA). The results were analyzed using BD FACS Diva software version 7 (BD Biosciences, East Rutherford, USA). The fluorescence of the stained cells was measured using BD LSR Fortessa (Becton Dickinson, Franklin Lakes, NJ, USA). The cells were excited at 488 nm, and the emission was measured at the 710/50 channel (PerCP-Cy5.5) to minimize the contribution of the MB signal. The events were gated to exclude debris and cell aggregates, and cell cycle analysis was performed using FlowJo v.10.

## Figures and Tables

**Table 1:** The actual MB concentration (µM, highlighted in bold) in gels according to MTT, ROS and cytofluorimetry analysis. The information about the samples is given in the table in Figure 1B.

Sample	CSS Concentration, µM					
	20	40	60	80	150	300
0	**0**	**0**	**0**	**0**	**0**	**0**
5	**0.05**	**0.1**	**0.2**	**0.27**	**0.5**	**1**
9	**0.24**	**0.49**	**0.96**	**1.29**	**2.38**	**4.76**
11	**0.5**	**1**	**2**	**2.7**	**5**	**10**
15	**2.4**	**4.9**	**9.6**	**12.9**	**23.8**	**47.6**
17	**5**	**10**	**20**	**27**	**50**	**100**

**Table 2:** The flow cytometric analysis of DNA content in SiHa cells treated by CSMBGs. The concentration of the samples is 30 µM. The information about the samples is given in the table in Figure 1B.

Sample	SubG0–G1, % No Irradiation/Irradiation	G0–G1, % No Irradiation/Irradiation	S, % No Irradiation/Irradiation	G2-M, % No Irradiation/Irradiation
0—no MB	17/11	68/68	4/6	11/15
5	4/3	81/74	5/9	10/14
9	4/2	73/74	9/7	14/17
11	4/2	76/76	8/6	12/16
15	8/3	78/77	4/6	10/14
17	8/3	81/77	4/6	7/14

**Table 3:** The flow cytometric analysis of DNA content in HaCat cells treated by CSMBGs. The concentration of the samples is 30 µM. The information about the samples is given in the table in Figure 1B.

Sample	SubG0–G1, % No Irradiation/Irradiation	G0–G1, % No Irradiation/Irradiation	S, % No Irradiation/Irradiation	G2-M, % No Irradiation/Irradiation
0—no MB	86/8	13/70	0.5/5	0.5/17
5	4/4	61	16/6	19/18
9	4/4	56/67	22/9	18/20
11	18/8	56/57	11/12	15/23
15	55/35	43/43	1/8	1/14
17	40/10	55/68	2/8	3/14

## Data Availability

All data and materials are available on request from the corresponding author. The data are not publicly available due to ongoing research using a part of the data.
